# Limited contemporary gene flow and high self-replenishment drives peripheral isolation in an endemic coral reef fish

**DOI:** 10.1002/ece3.584

**Published:** 2013-04-29

**Authors:** Martin H van der Meer, John B Horne, Michael G Gardner, Jean-Paul A Hobbs, Morgan Pratchett, Lynne van Herwerden

**Affiliations:** 1 Molecular Ecology and Evolution Laboratory, Australian Tropical Sciences and Innovation Precinct, James Cook UniversityTownsville, 4811, Australia; 2 School of Marine and Tropical Biology, James Cook UniversityTownsville, 4811, Australia; 3 ARC Centre of Excellence for Coral Reef Studies, James Cook UniversityTownsville, 4811, Australia; 4 School of Biological Sciences, Flinders UniversityGPO Box 2100, Adelaide, 5001, South Australia, Australia; 5 Evolutionary Biology Unit, South Australian MuseumAdelaide, 5000, South Australia, Australia; 6 The Oceans Institute and School of Plant Biology, University of Western Australia35 Stirling Highway, Crawley, 6009, Australia; 7 Australian Institute of Marine SciencePerth, 6009, Western Australia, Australia; 8 Centre for Sustainable Tropical Fisheries and Aquaculture, James Cook UniversityTownsville, 4811, Australia

**Keywords:** *Chaetodon*, coral reefs, extinction risk, Lord Howe Island, marine dispersal, Norfolk Island

## Abstract

Extensive ongoing degradation of coral reef habitats worldwide has lead to declines in abundance of coral reef fishes and local extinction of some species. Those most vulnerable are ecological specialists and endemic species. Determining connectivity between locations is vital to understanding recovery and long-term persistence of these species following local extinction. This study explored population connectivity in the ecologically-specialized endemic three-striped butterflyfish (*Chaetodon tricinctus*) using mt and msatDNA (nuclear microsatellites) to distinguish evolutionary versus contemporary gene flow, estimate self-replenishment and measure genetic diversity among locations at the remote Australian offshore coral reefs of Middleton Reef (MR), Elizabeth Reef (ER), Lord Howe Island (LHI), and Norfolk Island (NI). Mt and msatDNA suggested genetic differentiation of the most peripheral location (NI) from the remaining three locations (MR, ER, LHI). Despite high levels of mtDNA gene flow, there is limited msatDNA gene flow with evidence of high levels of self-replenishment (≥76%) at all four locations. Taken together, this suggests prolonged population recovery times following population declines. The peripheral population (NI) is most vulnerable to local extinction due to its relative isolation, extreme levels of self-replenishment (95%), and low contemporary abundance.

## Introduction

Coral reef fishes have evolved in a close relationship with coral reef habitats to produce the most diverse vertebrate communities on earth (Bellwood 1996; Wood 1999; Bellwood and Wainwright 2002; Bellwood et al. 2010). However, coral reef habitats are coming under increasing pressure, facing a multitude of impacts including destructive and excessive fishing, sedimentation, pollution, disease, coral bleaching, ocean warming, and acidification (Hoegh-Guldberg 1999; Hughes et al. 2003; Bellwood et al. 2004). These disturbances have combined to cause sustained and ongoing declines in the abundance of corals on reefs worldwide (e.g., Gardner et al. 2003; Bellwood et al. 2004) with approximately 20% of the world's coral reefs recently destroyed and a further 50% in decline (Wilkinson 2002); whilst coral cover on the Great Barrier Reef has halved in the last 27 years (De'ath et al. 2012). Given their strong reliance on live coral habitats, the abundance and diversity of reef fishes invariably declines with severe and/or prolonged declines in coral cover (Jones et al. 2004; Graham et al. 2006; Wilson et al. 2006; Pratchett et al. 2008). Extensive coral loss has resulted in the local extinction of some coral reef fishes, particularly those species that rely on live coral (Kokita and Nakazono 2001; Graham et al. 2006; Pratchett et al. 2008). Local extinction of coral dependent fishes are likely to increase if major disturbances that cause acute and extensive coral loss, such as coral bleaching, increase in incidence, as predicted (Hoegh-Guldberg 1999; Sheppard 2003).

In terrestrial habitats, endemic species (particularly on isolated islands) typically have higher rates of extinction and lower genetic diversity (Frankham 1997; Whittaker and Fernández-Palacios 2007). Coral reef fish communities on isolated islands tend to have a high proportion of endemics (Jones et al. 2002), and account for some of the most recent fish extinctions (Dulvy et al. 2003). Endemic species may be particularly vulnerable to widespread disturbances with their inherent small geographical range and small population size (Gaston 1998). This risk of extinction is further increased if endemic species have specific dietary (Pratchett et al. 2006; Graham 2007) or specialist habitat (Munday 2004; Wilson et al. 2006, 2008) requirements. The ability for coral dependent fishes to recover from local extinction will be dependent on the regeneration of their coral resources and larval replenishment from distant locations as assessed by gene flow. Thus, there is an urgent need to understand gene flow between, and genetic diversity at, locations inhabited by endemic reef fishes for ongoing monitoring and conservation, and to determine their recolonization ability and resilience.

To thoroughly understand gene flow it is important that both evolutionary and contemporary levels of gene flow are determined (i.e., at various time and spatial scales; Palstra et al. 2007) as some reef fish studies have shown discrepancies, up to an order of magnitude difference, in gene flow over these different time/spatial scales (i.e., high evolutionary but limited contemporary gene flow: Evans et al. 2010; Harrison et al. 2012; van der Meer et al. 2012a). Although determining levels of gene flow is important, of equal importance is conserving genetic diversity. Conserving genetic diversity is an International Union for Conservation of Nature (IUCN) priority (McNeely et al. 1990) for at least two reasons: (i) it provides the raw material for natural selection to act on over evolutionary (Johannesson and Andre 2006) and contemporary time scales (Bell and Okamura 2005); and (ii) low genetic diversity increases the risk of inbreeding depression (Reed and Frankham 2003).

Large data sets of highly polymorphic msatDNA loci (nuclear microsatellites) produced by next generation sequencing (e.g., Gardner et al. 2011) and advancements in statistical techniques (e.g., Pritchard et al. 2000; Beerli and Felsenstein 2001; Wilson and Rannala 2003; Excoffier et al. 2005; Jombart et al. 2010) have increased the sophistication of population genetic studies. However, to date, few such studies have been able to sample all existing locations across a species limited range. Unsampled “ghost” locations can affect key demographic estimates (i.e., population size, genetic diversity, migration rate; Beerli 2004). Here we investigate patterns of gene flow and measure population genetic diversities in an ecological specialist reef fish, the endemic three-striped butterflyfish (*Chaetodon tricinctus*), by complete sampling across its four geographically isolated locations (all found within Australian waters): Middleton Reef − MR, Elizabeth Reef − ER, Lord Howe Island − LHI, and Norfolk Island − NI.

The three-striped butterflyfish is an endemic to the LHI region (Randall 1976). This region is a hotspot for endemic coral reef fishes (Marine Parks Authority 2010) ranking fifth in the Indo-Pacific for percent endemism (7.2%, Randall 1998). Marine Protected Areas (MPAs) have been established to conserve reef fishes at three of these locations (MR, ER, LHI), but no protection exists at NI. This is an ideal study system as reef fishes occur on only four discrete islands/reefs that are separated by deep ocean water. Thus, connectivity of reef fish populations across the four locations is restricted to oceanic dispersal of pelagic larvae over known distances (e.g., 45–600 km).

Previous research on another endemic species in this system, the McCulloch's anemonefish (*Amphiprion mccullochi*), revealed limited contemporary gene flow between ER, MR, and LHI (van der Meer et al. 2012a,b). However, anemonefish have the shortest pelagic larval duration (PLD) of reef fishes (11–17 days: Victor 1986; Thresher et al. 1989; Wellington and Victor 1989; Victor and Wellington 2000) and their self-recruitment to natal areas has been well documented (Jones et al. 2005; Planes et al. 2009). While McCulloch's anemonefish provide a test of population connectivity in reef fishes at the lower limit of dispersal potential within the LHI region, determining the connectivity of reef fishes in general requires examining species from a common group with PLD's more typical of reef fish (20–50 days). Butterflyfishes (Chaetodontidae) are one of the 10 common families of fishes that are characteristic of coral reefs (Bellwood and Wainwright 2002). The PLD of *C. tricinctus* (mean = 35 days; M. van der Meer & J.-P. A Hobbs, unpublished data), is typical of butterflyfishes (26–53; e.g., Brothers et al. 1983; Brothers and Thresher 1985) and many other reef fishes. *C. tricinctus* is also one of the 41 butterflyfish species that feed directly on scleractinian corals (Cole et al. 2008; Rotjan and Lewis 2008). Thus, *C. tricinctus* provides a test of population connectivity in a common group of reef fishes, that is closely associated with coral reefs, and with a dispersal potential typical of most reef fishes.

*Chaetodon tricinctus* faces a higher risk of extinction as a consequence of its small geographic range, compared to its closest relatives *C. bennetti, C. plebeius,* and *C. trifascialis* (Bellwood et al. 2010), which are distributed widely throughout the Indo-Pacific (Allen et al. 1998). Moreover, *C. tricinctus* feeds exclusively on live corals (Kuiter 1996) and is mostly found in close association with corals of the genus *Acropora* (Hobbs et al. 2009). The abundance of *C. tricinctus* is positively linked to the abundance of *Acropora* spp., indicating that a loss of this coral could cause decreases in abundance and potential local extinction of *C. tricinctus* (Hobbs et al. 2009). The global abundance of *C. tricinctus* is likely to be much smaller than its widespread congenerics, and if it cannot alter its diet following coral loss, then these factors will compound upon its small geographic range and greatly increasing its vulnerability to local and possibly global extinction. Dramatic declines in abundance of several other butterflyfishes have occurred following extensive coral loss (Syms 1998; Pratchett et al. 2006), but some of the most vulnerable species have been spared from extinction due to their large geographic range (Lawton et al. 2011). Given that *C. tricinctus* exists at a few isolated locations and may be particularly vulnerable to local extinction, there is an obvious need to determine patterns of population connectivity and replenishment for this species.

The aims of this study were threefold: (i) to determine patterns and levels of gene flow between locations using mtDNA (mitochondrial DNA) and msatDNA; (ii) to estimate levels of self-replenishment (as a proxy for realized self-recruitment) and recent migration; and (iii) to measure population genetic diversities at all locations as an indicator of potential resilience of populations to environmental change and extinction.

## Materials and Methods

We applied a range of frequency and Bayesian based molecular tools to establish mtDNA and msatDNA levels of phylogenetic and population genetic structure. This resulted in a comprehensive understanding of gene flow in this study system and together these tools provided a comprehensive view of dispersal (Leis et al. 2011). However, due to the large number of analyses, we present only methods related to this study below, whilst general Materials and Methods (i.e., genetic and laboratory techniques and, in-depth mt and msatDNA analyses) are presented in van der Meer et al. (2012a,b,c). Fin clip sample sizes ranged from 21 to 31: MR (*n* = 30), ER (*n* = 31), LHI (*n* = 26), and NI (*n* = 21). We used a large number of polymorphic microsatellite loci (*n* = 20) and sampled all known locations, to compensate for the small sample sizes used in this study (see Selkoe and Toonen 2006). Furthermore, we recognize that our estimates for “self-replenishment” inferred indirectly from genetic markers are merely a proxy for self-recruitment, which is typically assessed using more direct methods (e.g., natural or artificial otolith tags), such as those used by Swearer et al. (1999), Jones et al. (2005), and Almany et al. (2007). Nevertheless, direct approaches are not feasible for our study species, without negatively impacting populations, due to the large sample sizes typically required for such parentage-based studies. Therefore, we believe that our indirect estimates of self-replenishment represent the best possible substitute for realized self-recruitment obtainable for this species.

### Ethics statement

The main aim of this study was to determine gene flow between and genetic diversity at isolated locations using the endemic three-striped butterflyfish (*C. tricinctus*) as a model organism. Fin clip samples were obtained from fishes of adult size (>100 mm total length) either by spearfishing or by anesthetizing fish with clove oil, which were fin clipped in situ and released alive (Permit Numbers: LHIMP08/R01, 003-RRRWN-110,211-02, P11/0035-1.0, LHIMP/R/2010/012; Animal ethics: A1605).

### Study system

**Figure 1:**
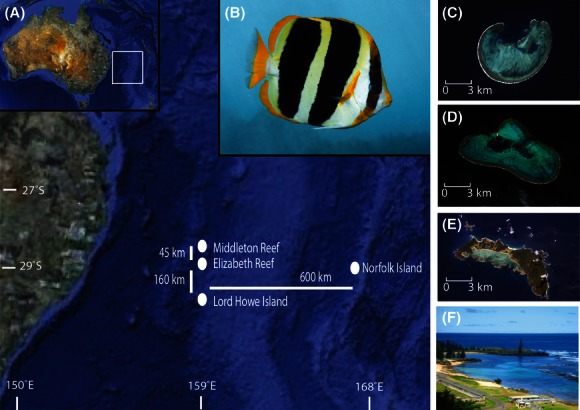
Location maps and focal species. (A) Google Earth image of eastern Australia showing Middleton Reef (MR), Elizabeth Reef (ER), Lord Howe Island (LHI), and Norfolk Island (NI) in the South West Pacific Ocean. (B) *Chaetodon tricinctus* swimming in the open (Photo courtesy of Justin Gilligan). Aerial photographs of MR (C); ER (D), LHI (E), and NI (F; the bay measures 1 km in length)

Throughout this study the three locations MR, ER, and LHI are collectively referred to as the “western region” because they occur on the same geographic feature (Lord Howe Island Rise – remnants of volcanoes estimated to be 6.7 Ma, McDougall et al. 1981), are relatively close to each other (Fig. 1) and all locations support high abundances of *C. tricinctus* (Choat et al. 2006; Hobbs et al. 2009). In contrast, NI is referred to as the “peripheral location” for *C. tricinctus,* because it is the only location situated on a separate geographic feature (Norfolk Island Rise – remnant of a volcano estimated to be 2.3–3.05 Ma, Jones and McDougall 1973), it is isolated by more than 600 km from the western region (Fig. 1) and has relatively low abundance (a total population size estimated to be less than 30 individuals – authors unpublished data).

### Gene flow between locations – mtDNA

#### mtDNA phylogenetic analysis

mtDNA Cytochrome b (cyt b) sequence data were obtained from GenBank for the following three most closely related species which acted as out-groups: *C. trifascialis* (FJ167707.1), *C. plebius* (AF108602.1), and *C. bennetti* (FJ167686.1) based on the findings of Bellwood et al. (2010). jModeltest (Posada 2008) identified an TrN + G model under Akaike Information Criterion with gamma = 0.759. The three most commonly used phylogenetic analyses: Maximum Likelihood (ML), Maximum Parsimony (MP), and Bayesian Inference (Mr Bayes – MB and BEAST) were performed on the aligned mtDNA sequence data as described in van der Meer et al. (2012a,b). This was done to identify any underlying evolutionary partitions in the data, based on the use of rigorous analytical tools. A Minimum Spanning Tree (MST) was generated based on output obtained from ARLEQUIN 3.5 (Excoffier et al. 2005) to explicitly identify shared haplotypes between *C. tricinctus* from the four locations.

#### Quantifying the level of mtDNA gene flow

mtDNA migration rates and effective population sizes of *C. tricinctus* were estimated between or within each of the four locations using MIGRATE-n 2.4.3 (http://popgen.sc.fsu.edu/Migrate-n.Html; Beerli and Felsenstein 2001; Beerli 2004). We tested a combination of various migration priors (*F*_st_ and own: isolation-by-distance), custom-migration models (Stepping-stone, Island-n, and variable Theta only) and a geographic matrix – all with a constant mutation rate. A Log Maximum-Likelihood analysis (Ln ML) selected a migration prior (*F*_st_), custom-migration model (migration model with variable Theta), constant mutation rate with an F84 mutation model, migration rate parameters (Theta and M to a maximum of 1 and 15,000, respectively), and a Bayesian analysis, using a heating search strategy of one long chain that sampled every 20th of 60 k sampled trees and applied a 20 k iteration burn-in. All parameters converged and fell within the 90% CI yielding values for *θ* and M (mutation-scaled migration rate) per location.

### Gene flow between locations – msatDNA

#### Patterns of gene flow (msatDNA)

Three molecular analytical tools were used to establish spatial population partitioning in msatDNA: (i) Discriminant Analysis of Principal Components (DAPC; Jombart et al. 2010) uses allelic states to discriminate between the four locations, yielding scatterplots of discriminant functions based on the spatial distributions of microsatellite genotypes. DAPC also provided posterior probabilities of population assignments for each individual; (ii) a likelihood-based assignment method was used in GeneClass2 (Paetkau et al. 1995, 2004; Piry et al. 2004) to determine significant interlocation gene flow and (iii) STRUCTURE V2.3 (Pritchard et al. 2000; Hubisz et al. 2009) placed individuals into clusters that minimize Hardy–Weinberg Equilibrium (HWE) and can be used to identify contemporary gene flow between the four locations. To determine the “best value” for *K*, we followed the method suggested by Pritchard et al. (2000), which involved comparing mean log-likelihoods penalized by one-half of their variances (see Hubisz et al. 2009). The final run consisted of an Admixture model with 2 M iterations and a 100 k iteration burn-in.

#### Quantifying the level of msatDNA gene flow

Contemporary migration rates and effective population sizes of *C. tricinctus* were tested and estimated between each of the four locations) using MIGRATE-n 2.4.3 as above. We set datatype to Microsatellite (a simple electrophoretic ladder model), migration prior (*F*_st_), custom-migration model (migration model with variable Theta), constant mutation rate with a stepwise mutation, migration rate parameters (Theta and M to a maximum of 10 and 20, respectively), and a Bayesian analysis, using a heating search strategy of one long chain that sampled every 20th of 60 k sampled trees and applied a 20 k iteration burn-in. All parameters converged and fell within the 90% CI yielding values for θ and M (mutation-scaled migration rate) per location.

#### Inferred levels of self-replenishment and recent migration

This study did not sample new butterflyfish recruits in order to determine self-recruitment as in Jones et al. (2005). However, we used BAYESASS v3 (Wilson and Rannala 2003), a program specifically designed for population genetic studies that estimates recent migration rates (past 2–3 generations) between populations (or locations). Conversely, this program also has the ability to estimate any individuals not migrating (i.e., self-replenishing). We used BAYESASS v3 to estimate both self-replenishment (as a proxy for realized self-recruitment) and recent migration between locations; with a Markov chain Monte Carlo (MCMC) chain, consisting of a total of 11 M steps with a 2 M step burn-in; prior values for migration rate, allele frequency, and inbreeding coefficient were specified as 0.5, 0.6, and 0.6, respectively. These priors were selected because they gave acceptance rates within the 20–40% range showing convergence of the MCMC (Faubet et al. 2007). Ten independent runs separately assessed convergence of the MCMC (i.e., priors fell within the 20–40% range suggesting convergence) in order to evaluate the consistency of results obtained from these inferences.

### Population genetic diversities

Molecular diversity indices for mtDNA (haplotype diversity, *h*; nucleotide diversity, *π*) and for msatDNA (genetic diversity, *gd*) were estimated in ARLEQUIN 3.5 (Excoffier et al. 2005). Haplotype and nucleotide diversities of the data were interpreted as either low with specified cut-off values of *h* and *π* (%) <0.5 or high if values of *h* and *π* (%) were >0.5 (Grant and Bowen 1998).

## Results

### Synopsis

**Figure 2:**
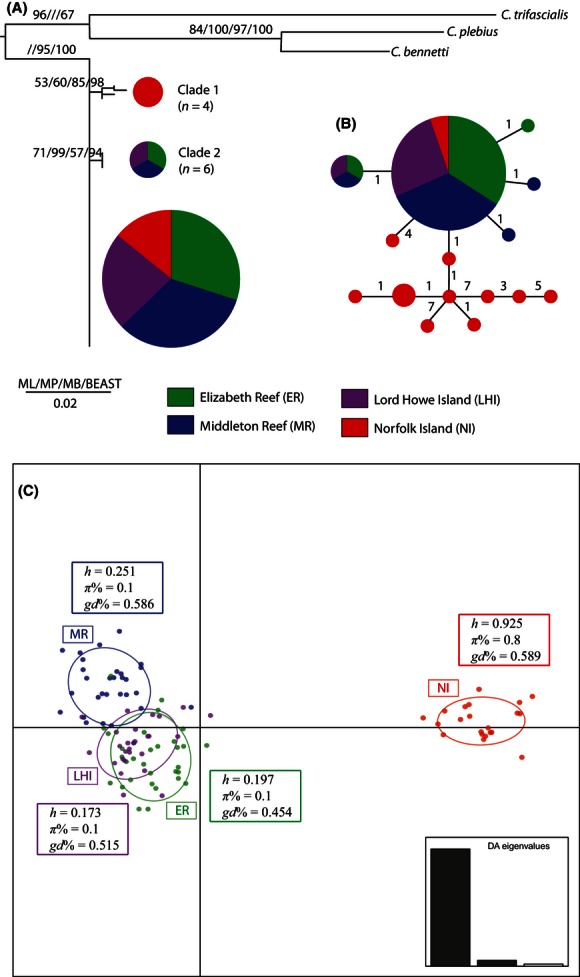
mt and msatDNA *Chaetodon tricinctus* analyses. (A) A phylogram of mtDNA (cyt b) sequences from 97 *C. tricinctus* individuals from Middleton Reef, Elizabeth Reef, Lord Howe Island, and Norfolk Island. This represents the best ML tree from 10 individual analyses. Numbers on branches indicate support for each clade, based on ML, MP, MB, and BEAST analyses. (B) Haplotype minimum spanning tree (MST) with number of substitutions between haplotypes indicated on connectors. Different fills represent each of the four locations as shown on the key to the figure, and (C) Scatterplots of the discriminant analysis of principal components of the microsatellite data for four *C. tricinctus* locations using geographic sample site as priors for genetic clusters. Individual genotypes appear as dots surrounded by 95% inertia ellipses. Eigenvalues show the amount of genetic information contained in each successive principal component with *X* and *Y* axes constituting the first two principle components, respectively. Boxes indicate haplotype (*h*), nucleotide (*% π*), and genetic diversity (*gd*) indices for *C. tricinctus*.

**Figure 3:**
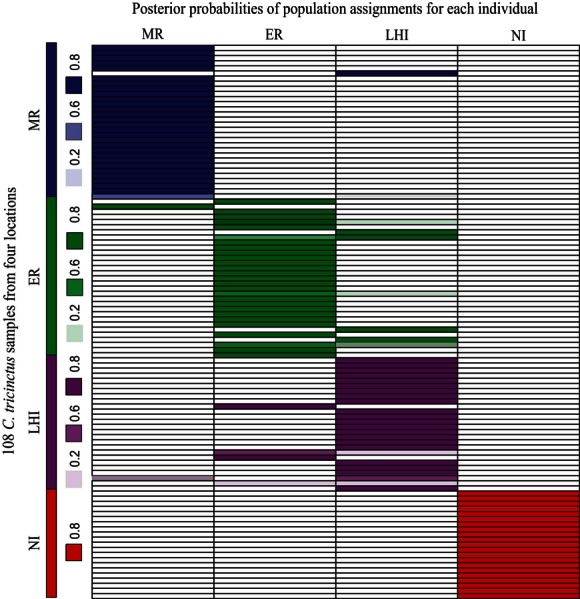
Posterior probability of assignment of each individual genotype to four *Chaetodon tricinctus* populations as indicated by DAPC. The names of the possible assignment populations are given on the *x*-axis. 108 genotypes are listed on the *y*-axis, along with the population from which they were sampled. Colored bars correspond to a 0.2–0.8 probability of assignment to a given population.

Two hundred and eighty-three base pairs of mtDNA (cyt b) were resolved for 97 *C. tricinctus* individuals; with a total of 15 polymorphic sites, of which three were parsimony informative. One small clade, Clade 1 (*n* = 4) contained exclusively individuals from the peripheral location. The other, Clade 2 (*n* = 6), comprised of an equal frequency of all three western locations (i.e., MR, ER, LHI; Fig. 2A). A MST identified 15 haplotypes in total, one of which was observed at high frequencies representing 82% (*n* = 80) of all individuals, and 12 of which were unique to single fish only in the sample examined here, nine of which were from the peripheral location (Fig. 2B). Mt and msatDNA Analysis of Molecular Variance (AMOVA) and pairwise *F*_st_ results indicate that there is population genetic differentiation between the western locations and the peripheral location, but there is no population genetic differentiation within the western region (i.e., when MR, ER, LHI are grouped). Haplotype and genotype diversities were low (<0.5) within the western region, but high (>0.5) at the peripheral location (Figs. 3 and S1). Genotypic diversity (*gd*), in contrast, was high at three of the four locations, ER being the exception. Detailed genetic diversity, AMOVA, summary statistics, pairwise population comparisons, and locus by locus AMOVA can be found in Supporting Information (S1, S2, S3, S4, and S5, respectively).

### Summary statistics

Heterozygote excess was evident from a negative inbreeding coefficient (*F*_IS_; Table S1); although this was not significant between the four locations. The peripheral location had the most private alleles, 25 across twenty loci, while the remaining three locations ranged from 6 to 15 private alleles across all loci (Table S2). Of the 20 msatDNA loci: (i) significant single locus departures from HWE were detected in 11 of 80 tests at the location level before False Discovery Rate (FDR) correction and nine afterwards (ER: Ct4, Ct23, Ct24; MR: Ct16; NI: Ct3, Ct13, Ct17, Ct23, Ct24; Table S2), similarly, six single locus HWE departures were detected before and after FDR when all locations were considered (Table S2); (ii) null alleles were identified in MR (Ct18) and NI (Ct10, Ct16); and (iii) of the 212 locus by locus exact tests of linkage disequilibrium, 13 were significant before and 10 after FDR correction (Benjamini and Hochberg 1995). Loci that were not in HWE in more than one location (Ct23, Ct24) and had null alleles (Ct10, Ct16, Ct18) were not used in subsequent analysis (ARLEQUIN, STRUCTURE, and MIGRATE-n) and, loci in linkage disequilibrium at all sites (Ct17, Ct21, Ct23) were not used in subsequent analysis (ARLEQUIN, STRUCTURE, BAYESASS). Thus 13 loci were used in the ARLEQUIN and STRUCTURE analyses, 15 loci were used in the MIGRATE -n analysis and 17 loci in the BAYESASS analysis.

### Gene flow between locations – mtDNA

*Patterns and levels of gene flow* based on an mtDNA AMOVA indicated significant genetic variation (65.77%) within locations, Φ_st_ = 0.342 (*P* < 0.001, Table S3). This was due to the peripheral location mtDNA pairwise *F*_st_ differentiation from all three western locations (pairwise *F*_st_ = 0.190 to 0.221, *P* < 0.001; Table S4). Whilst there was no genetic differentiation among the three western locations (pairwise *F*_st_ = −0.032 to −0.023, *P* = 0.865 to 0.991; Table S4). A single regional partition was also suggested between the western region and the peripheral location, explaining 36.45% of the genetic variation, but this was not significant (Φ_ct_ = 0.365, *P* = 0.250; Table S3).

**Figure 4:**
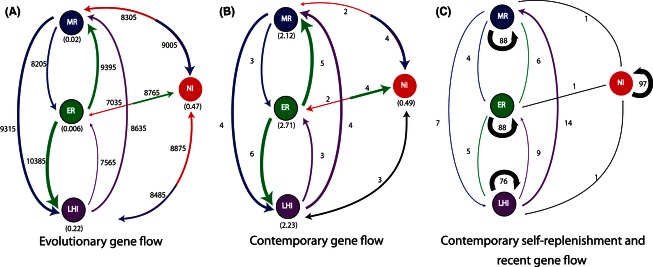
Migration rates among *Chaetodon tricinctus* locations. The thickness of the line is directionally proportional to the number of migrants (M) and the line colors indicate the predominant direction of gene flow. Population size (θ, within parentheses) is also shown for each location. (A) Migrate-n evolutionary gene flow (mtDNA), (B) Migrate-n contemporary gene flow (msatDNA), and (C) BAYESASS analysis of self-replenishment and recent migration rates (msatDNA) shown as a percentage.

*Quantifying mtDNA gene flow* using Migrate-n indicated high levels of mtDNA gene flow between all locations, with M values ranging from 7035 to 10,385 (Fig. 4A).

### Gene flow between locations – msatDNA

*Patterns and levels of gene flow* based on an msatDNA AMOVA indicated significant structure in five (of 20) locus by locus analyses corrected for null allele frequency (Φ_st_ = 0.001 to 0.368, *P* < 0.05; Table S5), in six (of 20) locus by locus analyses corrected for standardized location differentiation (Φ_st_ = 0.004 to 0.852, *P* < 0.05; Table S5) and in the global AMOVA as a weighted average over all microsatellite loci (Φ_st_ = 0.046, *P* < 0.001; Table S3), with 95.39% of the genetic variation existing within locations. Raw msatDNA pairwise *F*_st_ comparisons showed very low to moderately significant genetic partitioning between the western locations and the peripheral location (*F*_st_ = 0.056 to 0.101, *P* < 0.001). In contrast, an Excluding Null Alleles (ENA) corrected msatDNA pairwise *F*_st_ value showed no significant genetic differentiation between any of the four locations as estimates of genetic differentiation between locations fell within 95% confidence intervals (*F*_st_ = 0.005 to 0.084, *P* > 0.05; Table S4).

DAPC, GeneClass2, and STRUCTURE confirmed the presence of at least three distinct genetic populations corresponding to geographic location. DAPC partitioned *C. tricinctus* into the western region and the peripheral location (Fig. 2C). Using the four locations as *a priori* population criteria, DAPC assigned 58–100% of all individuals to the location from which they were sampled (assignment per population, ER = 74%, MR = 90%, LHI = 58%, NI = 100%; Fig. 3). Consistent with these assignments, with the allele frequencies and genotypic assignments, the 95% Genotypic Inertia Ellipses (GIE) for ER and LHI overlap, whilst the 95% GIE for MR does not overlap with either ER or LHI and the 95% GIE for NI occupy a distant area of multivariate space, along the *x*-axis, from all three western locations. Geographical structure in msatDNA data was confirmed by GeneClass2 analyses, where only 11 individuals were grouped with a location from which they were not sampled (MR = 1, ER = 7, LHIL = 3); thereby identifying four genetically differentiated populations. Similarly, four geographically partitioned populations were identified by STRUCTURE analyses, as the likelihood of the marginal posterior probability distribution was highest when *K* = 4.

#### Quantifying the level of msatDNA gene flow

Migrate-n indicated a few orders of magnitude lower levels of contemporary gene flow between locations when compared to mtDNA gene flow, with M values ranging from 2 to 6 (Fig. 4B).

#### Inferred levels of self-replenishment and migrant exchange

Despite weak genetic differentiation (*F*_st_) between locations within the western region, both DAPC and STRUCTURE partitioned the data into at least three distinct clusters. Used together, these programs are likely to be better than *F*_st_ values (Faubet et al. 2007) at determining the appropriateness of a dataset for BAYESASS because they extract more information from the genetic data than frequency-based fixation indices. However, it is important to note: (i) BAYESASS estimates of migration rates are accurate when migration rates are low, genetic differentiation between locations is not too low (*F*_st_ ≥ 0.05) and loci are in linkage equilibrium. Moreover, if the above-mentioned conditions are not met, then accurate estimates of migration rates are obtained only when migration rates are very low (*m* = 0.01) and genetic differentiation between locations is high (*F*_st_ ≥ 0.10; Faubet et al. 2007) and (ii) when estimates of migration rates fall below 10%, populations can probably be considered demographically independent from each other with no gene flow between locations (Waples and Gaggiotti 2006). Demographic independence is suggested for all location pairs except: LHI to MR (*m* = 14%) and possibly LHI to ER (*m* = 9%; Fig 4C). Conversely, high levels of self-replenishment (76–96%) were inferred at all four locations (Fig. 4C). This further indicates that in the short term, each population is predominantly sustained by self-replenishment rather than replenishment from distant populations.

### Population genetic diversities

*Chaetodon tricinctus* showed low haplotype diversity (*h*) and nucleotide diversity (% π) in all three western locations (*h* = 0.173 to 0.251, % π = 0.1), whilst the peripheral location had four- to eightfold higher *h* and % π (*h* = 0.925, % π = 0.8; Fig. 2C), respectively. Three of the four locations (MR, LHI, NI) had high genetic diversity (*gd* = 0.515–0.589), ER being the exception (*gd* = 0.454; Fig. 2C). Total haplotype, nucleotide, and genotypic diversities were low (*h* = 0.384, % π = 0.2, *gd* = 0.490; Table S1).

## Discussion

Understanding both time and spatial scales of gene flow and the levels of genetic diversity is vital to determine best practice management, maximize biodiversity conservation, and evaluate the capacity of coral reef fishes to recover should they become locally extinct. In this study, *C. tricinctus* was found to have (i) sufficient mtDNA gene flow connecting all locations within the western region, but low gene flow and consequent isolation of the peripheral population from the western locations; (ii) low msatDNA gene flow between all locations resulting in populations that are genetically differentiated; (iii) demographic dependence between LHI and MR (and possibly LHI and ER), yet high levels of inferred self-replenishment at all four locations; (iv) variable genetic diversities: low mtDNA genetic diversity at all three locations within the western region, but not at the peripheral location; and (v) high msatDNA genetic diversity at all four locations.

### Gene flow between locations – mtDNA

Monophyly was suggested for *C. tricinctus* with the exception of two clades, one of which consisted exclusively of the peripheral location. The lack of geographic population structure within the western region may result if a small number of recruits per generation maintain spatial genetic homogeneity (Shulman 1998; Planes 2002). Similar genetic homogeneity has been found in studies on the endemic Hawaiian butterflyfishes *Chaetodon multicinctus*, *C. miliaris,* and *C. fremblii* (Craig et al. 2010) and in numerous other coral reef fish species including the parrotfishes *S. frenatus* and *C. sordidus* (Dudgeon et al. 2000; Bay et al. 2004). In contrast, the genetic differentiation between the western region and the peripheral location likely results from limited gene flow due to geographic isolation (600 km of deep ocean separating each region) and complicated ocean currents (which are seasonally stronger or weaker flowing west to east along the East Australian Current and seasonally migrating north or south along the Tasman Front; Suthers et al. 2011). Such strong genetic breaks at peripheral locations has been demonstrated in other reef fishes, including two widespread coral reef snappers *Lutjanus kasmira* and *L. fulvus* (Gaither et al. 2010) and two widespread parrotfishes *Scarus psittacus* (Winters et al. 2010) and *Chlorurus sordidus* (Bay et al. 2004).

Despite high mtDNA gene flow between all locations, conventional statistics (AMOVA and pairwise *F*_st_) indicate that the three locations within the western region and the peripheral location are genetically differentiated. Although high mtDNA gene flow may provide some assistance to distant populations through recolonization following local extinctions and increasing genetic diversity (Hanski 1999; Jones et al. 2009), benefits to the maintenance of distant populations may be minimal, especially if combined with little or no contemporary gene flow on demographic time scales.

### Gene flow between locations – msatDNA

*Chaetodon tricinctus* showed contemporary genetic differentiation between all locations (with the possible exception of ER and LHI). The strong discrepancy between mtDNA and contemporary levels of gene flow in *C. tricinctus* is increasingly being documented in other coral reef fishes such as snappers *Lutjanus carponotatus* (Evans et al. 2010; Harrison et al. 2012) and *Lutjanus synagris* (Gold et al. 2011), coral trout *Plectropomus maculatus* (Evans et al. 2010; Harrison et al. 2012), and in the endemic LHI anemonefish *A. mccullochi* (van der Meer et al. 2012a). This “lack of congruence” between time scales may result from genetic homogeneity over evolutionary time scales (Shulman 1998; Planes 2002) compared to substantial amounts of self-recruitment over contemporary time scales (Swearer et al. 1999; Jones et al. 2005; Almany et al. 2007; Planes et al. 2009).

The estimation of contemporary gene flow is important for conservation because models predict that a few recruits per generation over evolutionary time scales will not sustain populations (Cowen et al. 2000, 2002). In light of this, MPAs are designed to be large enough for locations to sustain themselves and yet spaced close enough so that larvae produced within an MPA can potentially be exported to unprotected areas (see Halpern and Warner 2003; Shanks et al. 2003; Jones et al. 2005; Harrison et al. 2012). In the case of *C. tricinctus*, it is unlikely that the current MPAs in the western region will deliver any substantial recruitment to the peripheral location due to the geographic isolation and complicated ocean currents around the LHI and Norfolk Island Rise regions, and the high levels of larval retention possibly facilitated by natal homing (Botsford 2005; Hilborn et al. 2006). The high abundance of *C. tricinctus* at the western locations (Choat et al. 2006; Hobbs et al. 2009) reduces the likelihood of local extinction, while higher levels of contemporary gene flow, when compared to the peripheral location, are likely to facilitate recovery following population declines (or local extinction). Given the extremely small population size of *C. tricinctus* at NI (estimated to be less than 30 individuals), a slow recovery time is expected following population declines, due to intermittent pulse replenishment.

Less than 10% gene flow between populations suggests demographic independence (Waples and Gaggiotti 2006) and high levels of self-recruitment, which is vital for populations to persist (Hastings and Botsford 2006). However, trying to classify populations as “open” or “closed” may not be appropriate (Mora and Sale 2002; Largier 2003). Rather, populations that have 80% of the successful recruits generated internally, will take substantially longer to recover following local extinction than ones with only 20% self-recruitment (Miller and Shanks 2004) and should be considered *largely closed* or *largely open,* respectively. All locations appear demographically independent and may be considered largely closed. However, both MR and to a lesser extent ER, receives some gene flow from LHI, suggesting that the population at LHI is important for management and continued protection because it exports individuals to MR and ER. Levels of inferred self-replenishment found in *C. tricinctus* (≥76%) are highly similar to the estimated levels of self-recruitment in other congeneric butterflyfish in Papua New Guinea (PNG; Almany et al. 2007) and other island populations of coral reef fishes (Swearer et al. 1999; Jones et al. 2005; Planes et al. 2009). The consistency of results between the indirect methods of the present study and the direct methods of former studies suggest that self-replenishment can be used to approximate self-recruitment in coral reef fish populations, given a sufficient number of unlinked loci, high detectable levels of self-replenishment and no unsampled ghost locations. Moreover, estimates of self-replenishment in *C. tricinctus* tended to be slightly higher than estimates of self-recruitment in the above studies. It is unlikely that this difference is due entirely to methodological considerations, given that indirect genetic methods are thought to overestimate gene flow (Hellberg et al. 2002). Rather, high levels of self-recruitment in *C. tricinctus* might be inherent of its small geographic range and thus high self-recruitment is needed to sustain isolated populations in this system (Hobbs et al. 2011). Alternatively, studies sampling new recruits are also estimating self-recruitment during the postsettlement mortality period. Consequently, the genetic makeup of the recruit cohort that survives through to adulthood is changed creating a disparity between estimates of self-replenishment and self-recruitment.

### Population genetic diversities

*Chaetodon tricinctus* showed high mtDNA genetic diversity at the peripheral location and low diversity at the western locations. While msatDNA genetic diversity was high at three of the four locations (MR, LHI, NI), ER being the exception. Similar genetic diversities have been found in other coral reef fish using cytochrome b including the endemic Hawaiian butterflyfishes *Chaetodon fremblii*, *C. miliaris,* and *C. multicinctus* (Craig et al. 2010) and in the more widespread butterflyfishes *C. lunulatus, C. trifascialis,* and *C. trifasciatus* (Lawton et al. 2011; Montanari et al. 2011). Species with high genetic diversity may have some resilience to extinction as a decrease in genetic diversity is generally associated with decreased fitness (Hoelzel et al. 2002). Thus, the high overall genetic diversity at the peripheral location, resulting from pulse recruitment periodically bringing new genetic material into the population resident here and the occurrence of rare haplotypes (see below), is encouraging, as it may buffer a small, demographically isolated population against some impacts. However, the reverse patterns occur at the western locations, where the risk of extinction associated with low genetic diversity is counteracted by high population abundances. Of interest are the rare haplotypes seen only at the peripheral location that may represent either historical polymorphisms (a relic or refugium population) or mutation accumulation. Given the high abundance of *Acropora* at this location (authors unpublished data) but extremely low abundance of *C. tricinctus* individuals, it is likely that self-recruitment is limiting population numbers. If unique genetic diversity is a feature of NI populations of endemics within the region, then protecting these populations and the habitats they rely on is vitally important.

## Conclusion

Given the low contemporary gene flow between western and peripheral locations (and high self-replenishment) in *C. tricinctus*, the MPAs at the western region are of limited benefit to the unprotected peripheral location (NI). Therefore, the peripheral location requires some protective management strategies to conserve its genetically unique population of *C. tricinctus*. However, within the western region, LHI is an important source of gene flow to both MR and ER and as such, warrants continued MPA protection and monitoring. Similar patterns of gene flow between locations has also been found for the endemic Mcculloch's anemonefish, *A. mccullochi* (van der Meer et al. 2012a) and may be indicative of generalized patterns of gene flow of all endemics in the region. Given the importance of the LHI region as an endemic hotspot, determining patterns of gene flow across a number of endemic species with varying biological and ecological characteristics will be crucial for developing effective conservation management strategies.

## Supporting Information

**Table S1**. Sample sizes for mtDNA (cytochrome b, total *n* = 97).

**Table S2**. Summary statistics for twenty microsatellite loci (Ct2-24) from *Chaetodon tricinctus*.

**Table S3**. AMOVA analysis for (A) mtDNA (Cyt b) sequences from *Chaetodon tricinctus* structured into the westernmost location (MR, ER, LHI) versus the peripheral location (NI) and (B) global AMOVA weighted across all 20 microsatellite loci.

**Table S4**. Pairwise population structures (*F*_st_) generated for mtDNA (cytochrome b, *n* = 97) and for twenty microsatellite loci (*n* = 108) from four *Chaetodon tricinctus* locations showing raw and corrected *F*′_st_ for null allele frequencies.

**Table S5**. AMOVA fixation indices (*F*′_st_) for *Chaetodon tricinctus* across all locations surveyed.
