# Supplementary material for: Limited contemporary gene flow and high self-replenishment drives peripheral isolation in an endemic coral reef fish
Source: Ecol Evol. 2013 Apr 29;3(6):1653–66. doi: 10.1002/ece3.584 (PMC3686199; doi:10.1002/ece3.584)
Supplement: Supplementary file 1 [file ece30003-1653-SD1.doc]

Table S1: Sample sizes for mtDNA (cytochrome b, total *n* = 97)

|  | *n* (Cyt b) | *n_h_* | *h* | *π(%)* | *n* (msat) | *gd* | Ave N_a_ | P_a_ | H_o_ | H_e_ | F_is_ |
| --- | --- | --- | --- | --- | --- | --- | --- | --- | --- | --- | --- |
| All | 97 | 15 | 0.384 | 0.2 | 108 | 0.490 | 6.15 |  | 0.662 | 0.634 | -0.052 |
| ER | 29 | 3 | 0.197 | 0.1 | 31 | 0.454 | 6.35 | 11 | 0.699 | 0.656 | -0.041 |
| MR | 30 | 4 | 0.251 | 0.1 | 30 | 0.568 | 6.30 | 15 | 0.652 | 0.635 | -0.008 |
| LHI | 22 | 2 | 0.173 | 0.1 | 26 | 0.515 | 5.85 | 6 | 0.663 | 0.631 | -0.028 |
| NI | 16 | 11 | 0.925 | 0.8 | 21 | 0.589 | 6.10 | 25 | 0.670 | 0.646 | -0.036 |

cyt b, cytochrome b.

Number of samples (n), number of haplotypes (*n_h_*), haplotype diversity (*h*), nucleotide diversities (*π*) of cyt b for *Chaetodon tricinctus* from all locations. Sample sizes for msats (total n = 108), genetic diversity (*gd*), average number of alleles per locus (Na), observed number of private alleles (Pa), observed heterozygosity (H_o_), expected heterozygosity (H_e_) and the inbreeding coefficient (F_IS_) averaged over twenty-one microsatellite loci for four locations.
