# Supplementary material for: Limited contemporary gene flow and high self-replenishment drives peripheral isolation in an endemic coral reef fish
Source: Ecol Evol. 2013 Apr 29;3(6):1653–66. doi: 10.1002/ece3.584 (PMC3686199; doi:10.1002/ece3.584)
Supplement: Supplementary file 2 [file ece30003-1653-SD2.doc]

Table S2. Summary statistics for twenty microsatellite loci (Ct2-24) from *Chaetodon tricinctus*. Sample sizes (N), observed number of alleles (Na), observed number of private alleles (Pa), observed heterozygosity (H_o_), expected heterozygosity (H_e_), the average inbreeding coefficient (F_IS_), probability of departure from HWE (pHWE, Peakall and Smouse 2006) and significance of departure after FDR correction FDR (pFDR, Storey 2002) for each locus at all and each location (significance of departure in bold, p < 0.05).

| Population | Ct2 | Ct3 | Ct4 | Ct5 | Ct7 | Ct8 | Ct9 | Ct10 | Ct11 | Ct12 | Ct13 | Ct14 | Ct16 | Ct17 | Ct18 | Ct20 | Ct21 | Ct22 | Ct23 | Ct24 |
| --- | --- | --- | --- | --- | --- | --- | --- | --- | --- | --- | --- | --- | --- | --- | --- | --- | --- | --- | --- | --- |
| ***All (108)*** |  |  |  |  |  |  |  |  |  |  |  |  |  |  |  |  |  |  |  |  |
| N | 71 | 60 | 77 | 62 | 70 | 72 | 79 | 73 | 70 | 77 | 62 | 78 | 75 | 68 | 77 | 66 | 72 | 69 | 76 | 80 |
| Na | 4 | 6 | 8 | 6 | 11 | 8 | 6 | 6 | 11 | 5 | 11 | 7 | 9 | 8 | 5 | 10 | 16 | 10 | 6 | 5 |
| Pa | 1 | 1 | 2 | 1 | 4 | 4 | 1 | 3 | 2 | 1 | 2 | 1 | 4 | 3 | 1 | 2 | 3 | 2 | 2 | 2 |
| Ho | 0.423 | 0.400 | 0.675 | 0.629 | 0.986 | 0.694 | 0.709 | 0.521 | 0.857 | 0.558 | 0.823 | 0.705 | 0.600 | 0.721 | 0.403 | 0.758 | 0.833 | 0.826 | 0.868 | 0.175 |
| He | 0.465 | 0.400 | 0.650 | 0.688 | 0.777 | 0.631 | 0.640 | 0.524 | 0.758 | 0.586 | 0.824 | 0.745 | 0.574 | 0.722 | 0.420 | 0.812 | 0.882 | 0.857 | 0.658 | 0.217 |
| F_IS_ | 0.092 | 0.000 | -0.039 | 0.085 | -0.269 | -0.100 | -0.107 | 0.008 | -0.130 | 0.047 | 0.002 | 0.053 | -0.045 | 0.003 | 0.042 | 0.067 | 0.055 | 0.036 | -0.321 | 0.193 |
| pHWE | 0.092 | 0.765 | **0.000** | 0.251 | 0.040 | 0.926 | 0.770 | 0.877 | 0.995 | 0.943 | **0.000** | 0.646 | 0.150 | 0.715 | 0.663 | 0.565 | **0.000** | **0.003** | **0.000** | **0.000** |
| pFDR | 0.229 | 0.951 | **0.000** | 0.479 | 0.120 | 0.990 | 0.951 | 0.990 | 0.995 | 0.990 | **0.000** | 0.951 | 0.315 | 0.951 | 0.951 | 0.951 | **0.000** | **0.011** | **0.000** | **0.000** |
|  |  |  |  |  |  |  |  |  |  |  |  |  |  |  |  |  |  |  |  |  |
| ***ER (31****)* |  |  |  |  |  |  |  |  |  |  |  |  |  |  |  |  |  |  |  |  |
| N | 19 | 19 | 24 | 20 | 20 | 20 | 28 | 24 | 16 | 26 | 21 | 26 | 24 | 18 | 25 | 14 | 22 | 15 | 24 | 29 |
| Na | 3 | 5 | 7 | 6 | 9 | 4 | 5 | 5 | 7 | 5 | 11 | 6 | 5 | 5 | 4 | 7 | 14 | 9 | 5 | 4 |
| Pa | 0 | 0 | 1 | 1 | 3 | 0 | 0 | 1 | 0 | 1 | 2 | 0 | 2 | 0 | 0 | 1 | 1 | 1 | 1 | 2 |
| Ho | 0.474 | 0.421 | 0.708 | 0.750 | 1.000 | 0.650 | 0.714 | 0.583 | 0.813 | 0.577 | 0.905 | 0.692 | 0.708 | 0.778 | 0.600 | 0.786 | 0.773 | 0.867 | 0.917 | 0.103 |
| He | 0.514 | 0.357 | 0.713 | 0.681 | 0.764 | 0.625 | 0.679 | 0.588 | 0.752 | 0.577 | 0.864 | 0.702 | 0.577 | 0.702 | 0.473 | 0.770 | 0.870 | 0.849 | 0.661 | 0.162 |
| F_IS_ | 0.078 | -0.178 | 0.006 | -0.101 | -0.309 | -0.040 | -0.052 | 0.007 | -0.081 | 0.000 | -0.047 | 0.014 | -0.227 | -0.108 | -0.269 | -0.020 | 0.112 | -0.021 | -0.388 | 0.363 |
| pHWE | 0.655 | 0.999 | **0.001** | 0.668 | 0.893 | 0.389 | 0.897 | 0.465 | 0.807 | 0.643 | 0.096 | 0.582 | 0.776 | 0.567 | 0.597 | 0.559 | 0.137 | 0.155 | **0.000** | **0.000** |
| pFDR | 0.647 | 0.737 | **0.005** | 0.647 | 0.695 | 0.647 | 0.695 | 0.647 | 0.694 | 0.647 | 0.307 | 0.647 | 0.694 | 0.647 | 0.647 | 0.647 | 0.343 | 0.343 | **0.000** | **0.000** |

**Table S3** *(Continued)*

| Population | Ct2 | Ct3 | Ct4 | Ct5 | Ct7 | Ct8 | Ct9 | Ct10 | Ct11 | Ct12 | Ct13 | Ct14 | Ct16 | Ct17 | Ct18 | Ct20 | Ct21 | Ct22 | Ct23 | Ct24 |
| --- | --- | --- | --- | --- | --- | --- | --- | --- | --- | --- | --- | --- | --- | --- | --- | --- | --- | --- | --- | --- |
| ***MR (30)*** |  |  |  |  |  |  |  |  |  |  |  |  |  |  |  |  |  |  |  |  |
| N | 30 | 24 | 30 | 24 | 28 | 30 | 27 | 29 | 30 | 30 | 24 | 28 | 30 | 28 | 28 | 29 | 28 | 30 | 28 | 27 |
| Na | 3 | 5 | 7 | 5 | 7 | 7 | 6 | 4 | 9 | 4 | 8 | 7 | 7 | 7 | 4 | 9 | 13 | 8 | 4 | 3 |
| Pa | 1 | 1 | 1 | 0 | 1 | 3 | 1 | 1 | 0 | 0 | 0 | 1 | 2 | 2 | 1 | 0 | 1 | 0 | 0 | 0 |
| Ho | 0.300 | 0.417 | 0.633 | 0.583 | 0.964 | 0.733 | 0.741 | 0.586 | 0.833 | 0.533 | 0.667 | 0.750 | 0.567 | 0.643 | 0.214 | 0.793 | 0.857 | 0.833 | 0.929 | 0.185 |
| He | 0.383 | 0.444 | 0.618 | 0.694 | 0.654 | 0.654 | 0.628 | 0.499 | 0.767 | 0.563 | 0.778 | 0.736 | 0.553 | 0.719 | 0.339 | 0.822 | 0.889 | 0.846 | 0.675 | 0.230 |
| F_IS_ | 0.216 | 0.063 | -0.024 | 0.159 | -0.474 | -0.121 | -0.179 | -0.175 | -0.086 | 0.052 | 0.143 | -0.019 | -0.025 | 0.106 | 0.367 | 0.035 | 0.036 | 0.015 | -0.375 | 0.194 |
| pHWE | 0.544 | 0.821 | **0.047** | 0.446 | 0.280 | 0.950 | 0.993 | 0.532 | 0.983 | 0.307 | 0.945 | 0.254 | **0.000** | 0.728 | 0.004 | 0.860 | 0.006 | 0.438 | 0.002 | 0.621 |
| pFDR | 0.879 | 0.993 | 0.197 | 0.879 | 0.806 | 0.993 | 0.993 | 0.879 | 0.993 | 0.806 | 0.993 | 0.806 | **0.000** | 0.993 | 0.028 | 0.993 | 0.032 | 0.879 | 0.021 | 0.932 |
|  |  |  |  |  |  |  |  |  |  |  |  |  |  |  |  |  |  |  |  |  |
| ***LHI (26)*** |  |  |  |  |  |  |  |  |  |  |  |  |  |  |  |  |  |  |  |  |
| N | 22 | 17 | 23 | 18 | 22 | 22 | 24 | 20 | 24 | 21 | 17 | 24 | 21 | 22 | 24 | 23 | 22 | 24 | 24 | 24 |
| Na | 3 | 3 | 5 | 5 | 7 | 5 | 5 | 4 | 10 | 4 | 8 | 6 | 4 | 6 | 4 | 9 | 11 | 9 | 5 | 3 |
| Pa | 0 | 0 | 0 | 0 | 0 | 1 | 0 | 0 | 2 | 0 | 0 | 0 | 0 | 1 | 0 | 0 | 1 | 1 | 1 | 0 |
| Ho | 0.545 | 0.353 | 0.696 | 0.556 | 1.000 | 0.682 | 0.667 | 0.350 | 0.917 | 0.571 | 0.941 | 0.667 | 0.524 | 0.773 | 0.417 | 0.696 | 0.864 | 0.792 | 0.750 | 0.250 |
| He | 0.501 | 0.372 | 0.602 | 0.603 | 0.791 | 0.569 | 0.595 | 0.471 | 0.708 | 0.602 | 0.785 | 0.739 | 0.541 | 0.723 | 0.433 | 0.784 | 0.856 | 0.845 | 0.613 | 0.260 |
| F_IS_ | -0.089 | 0.051 | -0.155 | 0.079 | -0.264 | -0.198 | -0.121 | 0.257 | -0.294 | 0.051 | -0.198 | 0.098 | 0.031 | -0.069 | 0.038 | 0.112 | -0.008 | 0.063 | -0.224 | 0.037 |
| pHWE | 0.836 | 0.935 | 0.624 | **0.039** | 0.247 | 0.846 | 0.643 | 0.701 | 1.000 | 0.157 | 0.192 | 0.288 | 0.947 | 0.436 | 0.334 | 0.495 | 0.681 | 0.184 | 0.126 | 0.441 |
| pFDR | 0.987 | 0.994 | 0.920 | 0.410 | 0.741 | 0.987 | 0.920 | 0.920 | 1.000 | 0.672 | 0.672 | 0.756 | 0.994 | 0.842 | 0.779 | 0.866 | 0.920 | 0.672 | 0.672 | 0.842 |

**Table S3** *(Continued)*

| Population | Ct2 | Ct3 | Ct4 | Ct5 | Ct7 | Ct8 | Ct9 | Ct10 | Ct11 | Ct12 | Ct13 | Ct14 | Ct16 | Ct17 | Ct18 | Ct20 | Ct21 | Ct22 | Ct23 | Ct24 |
| --- | --- | --- | --- | --- | --- | --- | --- | --- | --- | --- | --- | --- | --- | --- | --- | --- | --- | --- | --- | --- |
| ***NI (21)*** |  |  |  |  |  |  |  |  |  |  |  |  |  |  |  |  |  |  |  |  |
| N | 17 | 21 | 20 | 21 | 21 | 20 | 21 | 17 | 20 | 17 | 21 | 20 | 17 | 21 | 20 | 20 | 21 | 20 | 16 | 21 |
| Na | 4 | 5 | 9 | 6 | 1 | 5 | 7 | 3 | 8 | 4 | 9 | 5 | 6 | 11 | 6 | 6 | 9 | 8 | 6 | 4 |
| Pa | 1 | 1 | 3 | 1 | 1 | 1 | 1 | 0 | 2 | 0 | 0 | 0 | 6 | 2 | 0 | 0 | 0 | 2 | 1 | 0 |
| Ho | 0.529 | 0.333 | 0.950 | 0.524 | 0.000 | 0.650 | 0.667 | 0.294 | 0.600 | 0.588 | 0.810 | 0.800 | 0.529 | 0.952 | 1.000 | 0.650 | 0.714 | 0.800 | 1.000 | 1.000 |
| He | 0.507 | 0.465 | 0.738 | 0.592 | 0.000 | 0.625 | 0.604 | 0.524 | 0.756 | 0.637 | 0.831 | 0.735 | 0.727 | 0.800 | 0.646 | 0.760 | 0.813 | 0.843 | 0.693 | 0.625 |
| F_IS_ | -0.044 | 0.283 | -0.288 | 0.115 | 0.000 | -0.040 | -0.103 | 0.439 | 0.207 | 0.076 | 0.026 | -0.088 | 0.271 | -0.190 | -0.547 | 0.145 | 0.121 | 0.050 | -0.442 | -0.601 |
| pHWE | 0.981 | **0.000** | 0.997 | 0.660 | na | 0.853 | 0.981 | 0.062 | 0.014 | 0.565 | **0.000** | 0.143 | **0.018** | 0.199 | 0.172 | 0.109 | 0.202 | 0.204 | **0.001** | **0.002** |
| pFDR | 0.997 | **0.000** | 0.997 | 0.836 | na | 0.997 | 0.997 | 0.168 | 0.053 | 0.767 | **0.000** | 0.298 | 0.057 | **0.298** | 0.298 | 0.259 | 0.298 | 0.298 | **0.006** | **0.01** |

|  |  |  |  |  |  |  |  |  |  |  |  |  |  |  |  |  |  |  |  |  |
| --- | --- | --- | --- | --- | --- | --- | --- | --- | --- | --- | --- | --- | --- | --- | --- | --- | --- | --- | --- | --- |
|  |  |  |  |  |  |  |  |  |  |  |  |  |  |  |  |  |  |  |  |  |
|  |  |  |  |  |  |  |  |  |  |  |  |  |  |  |  |  |  |  |  |  |
|  |  |  |  |  |  |  |  |  |  |  |  |  |  |  |  |  |  |  |  |  |
|  |  |  |  |  |  |  |  |  |  |  |  |  |  |  |  |  |  |  |  |  |
|  |  |  |  |  |  |  |  |  |  |  |  |  |  |  |  |  |  |  |  |  |
|  |  |  |  |  |  |  |  |  |  |  |  |  |  |  |  |  |  |  |  |  |
|  |  |  |  |  |  |  |  |  |  |  |  |  |  |  |  |  |  |  |  |  |
