# Supplementary material for: Limited contemporary gene flow and high self-replenishment drives peripheral isolation in an endemic coral reef fish
Source: Ecol Evol. 2013 Apr 29;3(6):1653–66. doi: 10.1002/ece3.584 (PMC3686199; doi:10.1002/ece3.584)
Supplement: Supplementary file 3 [file ece30003-1653-SD3.doc]

Table S3: AMOVA analysis for a) mtDNA (Cyt b) sequences from *Chaetodon tricinctus* structured into the westernmost location (MR, ER, LHI) vs the peripheral location (NI) and b) global AMOVA weighted across all twenty microsatellite loci.

| **Source of variation** | **Variance component** | **Percentage of variation** | **F-statistics fixation indices**  **(p-value)** |
| --- | --- | --- | --- |
| ***a) Location*** |  |  |  |
| Among groups | 0.147 | 36.45 | F_ct_ = 0.365  (*p* = 0.250) |
| Among populations  within locations | -0.009 | -2.22 | F_sc_ = -0.035  (*p* = 0.983) |
| Within populations | 0.266 | 65.77 | F_st_ = 0.342  (*p* < 0.001) |
| ***b) Microsatellite*** |  |  |  |
| Among groups | 0.24 | 3.53 | F_ct_ = 0.035  (*p* = 0.047) |
| Among populations  within locations | 0.07 | 1.07 | F_sc_ = 0.011  (*p* = 0.004) |
| Within populations | 6.46 | 95.39 | F_st_ = 0.046  (*p* < 0.001) |
