# Supplementary material for: Limited contemporary gene flow and high self-replenishment drives peripheral isolation in an endemic coral reef fish
Source: Ecol Evol. 2013 Apr 29;3(6):1653–66. doi: 10.1002/ece3.584 (PMC3686199; doi:10.1002/ece3.584)
Supplement: Supplementary file 4 [file ece30003-1653-SD4.doc]

Table S4. Pairwise population structures (F_st_) generated for mtDNA (cytochrome b, n = 97) and for twenty microsatellite loci (n = 108) from four *Chaetodon tricinctus* locations showing raw and corrected F΄_st_ for null allele frequencies.

Raw locations differentiation from microsatellite allele frequencies and associated *p*-values using Arlequin ver 3.5 (Excoffier et al*.* 2005). Corrected location differentiation for null allele frequencies at 95% CI (all *p*-values > 0.05) using the ENA correction of Chapuis and Estoup (2007). FDR correction of raw F΄ _st_ *p*-values in QVALUE (Storey 2002). The use of both p-values and Confidence Intervals (CI) is responsible for the discrepancy between the FST values. While the p-value is used to test if the sample is significant or not from zero (i.e. is the FST different from zero), the CI is used to construct confidence around the estimate [i.e.is there significant genetic differentiation (FST) between locations].Thus using the raw msatDNA data we find that the p-value is significant from zero. However, when we correct for null alleles (ENA), our confidence estimate suggests that there is no genetic differentiation (FST) between the locations.

|  | cyt b | | |  | Raw msat | | |  | Corrected msat | | |  |
| --- | --- | --- | --- | --- | --- | --- | --- | --- | --- | --- | --- | --- |
|  | ER | MR | LHI | NI | ER | MR | LHI | NI | ER | MR | LHI | NI |
| ER |  | 0.865 | 0.991 | **0.000** |  | 0.991 | 0.413 | **0.000** |  | p > 0.05 | p > 0.05 | p > 0.05 |
| MR | -0.023 |  | 0.991 | **0.009** | -0.040 |  | 0.991 | **0.000** | 0.011 |  | p > 0.05 | p > 0.05 |
| LHI | -0.032 | -0.031 |  | **0.009** | -0.030 | 0.001 |  | **0.000** | 0.005 | 0.006 |  | p > 0.05 |
| NI | 0.216 | 0.221 | 0.190 |  | 0.056 | 0.101 | 0.088 |  | 0.070 | 0.084 | 0.072 |  |
