# Supplementary material for: Limited contemporary gene flow and high self-replenishment drives peripheral isolation in an endemic coral reef fish
Source: Ecol Evol. 2013 Apr 29;3(6):1653–66. doi: 10.1002/ece3.584 (PMC3686199; doi:10.1002/ece3.584)
Supplement: Supplementary file 5 [file ece30003-1653-SD5.doc]

Table S5: AMOVA fixation indices (F΄_st_) for *Chaetodon tricinctus* across all locations surveyed

|  | Marker class and analysis | | |
| --- | --- | --- | --- |
|  | Raw msat | Msat corrected for  null allele freq. | Standardised msat  (F΄_st_) |
| Average | **0.046** | 0.040 | 0.113 |
| Ct2 | 0.001 | -0.001 | **0.004** |
| Ct3 | -0.006 | -0.004 | -0.017 |
| Ct4 | 0.000 | **0.004** | **0.006** |
| Ct5 | 0.019 | 0.025 | 0.068 |
| Ct7 | **0.397** | **0.368** | **0.852** |
| Ct8 | -0.001 | -0.002 | -0.005 |
| Ct9 | -0.006 | -0.005 | -0.014 |
| Ct10 | -0.005 | -0.006 | -0.017 |
| Ct11 | 0.003 | **0.005** | **0.013** |
| Ct12 | **0.052** | 0.036 | 0.091 |
| Ct13 | 0.016 | 0.011 | 0.073 |
| Ct14 | 0.011 | 0.018 | 0.070 |
| Ct16 | **0.035** | 0.040 | 0.091 |
| Ct17 | 0.024 | 0.019 | 0.077 |
| Ct18 | **0.077** | 0.059 | 0.130 |
| Ct20 | 0.001 | -0.005 | -0.016 |
| Ct21 | 0.001 | -0.002 | -0.017 |
| Ct22 | 0.006 | **0.001** | **0.007** |
| Ct23 | **0.043** | 0.039 | 0.117 |
| Ct24 | 0.184 | **0.127** | **0.222** |

Raw locations differentiation from microsatellite allele frequencies for each individual locus and as the average across all loci, locations differentiation corrected for null allele frequencies using the ENA correction of Chapuis & Estoup (2007) and standardized locations differentiation for and across all loci (F΄_st_). All values in bold are significant to the p<0.05 (i.e. 95% confidence interval).
